# 3D molecular structural modeling and characterization of indium phosphide via irregularity topological indices

**DOI:** 10.1186/s13065-024-01204-4

**Published:** 2024-05-16

**Authors:** Muhammad Salman, Asad Ullah, Shahid Zaman, Emad E. Mahmoud, Melaku Berhe Belay

**Affiliations:** 1 Department of Mathematics, University of Sialkot, Sialkot, 51310 Pakistan; 2 Department of Mathematical Sciences, Karakoram International University Gilgit, Gilgit, 15100 Pakistan; 3 Department of Mathematics and Statistics, Collage of Science, Taif University, P.O. Box 11099, 21944 Taif, Saudi Arabia; 4 Nanotechnology Center of Excellence, Addis Ababa Science and Technology University, P.O. Box 16417, Addis Ababa, Ethiopia; 5 Mathematics, Physics and Statistics Division, Addis Ababa Science and Technology University, P.O. Box 16417, Addis Ababa, Ethiopia

**Keywords:** Indium Phosphide, Crystal structure, Graph theory, Topological indices, Mathematical chemistry

## Abstract

Indium phosphide (InP) is a binary semiconductor composed of indium and phosphorus. It has a zinc blende crystal structure, which is a type of cubic lattice structure. This lattice is composed of indium and phosphorus atoms arranged in a lattice of cube-shaped cells, with each cell containing four indium atoms and four phosphorus atoms. This lattice structure is the same for all materials with a zinc blende crystal structure and is the most common type of lattice structure in semiconductors. Indium phosphide (InP) has several chemical applications. It is commonly used as a dopant in the production of semiconductors, where it helps control the electrical properties of the material. InP is also utilized in the synthesis various indium-containing compounds, which can have applications in catalysts and chemical reactions. Additionally, InP nanoparticles have been investigated for their potential use in biomedical imaging and drug delivery systems. The topological characterization of 3D molecular structures can be performed via graph theory. In graph theory, the connections between atoms are represented as edges and the atoms themselves are represented as nodes. Furthermore, graph theory can be used to calculate the topological descriptors of the molecule such as the degree-based and reverse degree-based irregularity toplogical indices. These descriptors can be used to compare the topology of different molecules. This paper deals with the modeling and topological characterization of indium phosphide $({\text{InP}})$ via degree-based and reverse irregularity indices. The 3D crystal structure of the InP is topologically modeled via partition of the edges, and derived closed form expressions for its irregularity indices. Our obtained results will be surely be helpful in investigating the QSPR/QSAR analysis as well as understanding the deep irregular behavior of the indium phosphide $({\text{InP}})$.

## Introduction

Chemical graphs, are graphs that represent the molecular and atomic structures including bonds and interactions, represent the fundamental framework of molecules and atoms [1–3]. Chemical graphs are often used in chemistry and biochemistry research, as well as in computer simulations of chemical processes [4].

The molecular/chemical graph refers to the representation of a molecular/chemical substance in the form of a graph [5, 6]. A molecular graph is a graphical representation of a molecule, where the atoms are represented by vertices and the chemical bonds by edges. The edges represent the interactions between the atoms, with each edge representing a single bond. The structure of the graph uses to determine the properties of the molecule, such as its shape and chemical properties. For example, the connectivity of graph used to determine the type of molecule (e.g. linear or cyclic), and the number of edges can be used to determine the number of chemical bonds in the molecule [7]. Degree based indices measure the number of edges that are connected to a vertex of a graph. These indices are a measure of the degree of connectivity of a vertex. The degree of a vertex is the number of edges incident to it. These indices are also referred to as degree centrality.

The degree based index of a graph can be used to describe the graph’s structure and the patterns of connectivity between its vertices [8]. It is a useful tool for analyzing the connectivity of networks, as well as for predicting the behavior of a given graph or network. Degree indices are also used to identify vertices or edges that are important for the overall structure of a graph or network. For example, in social networks, degree indices can be used to identify influential nodes. Additionally, degree indices can be used to measure the clustering of vertices, or to detect communities within a graph [9–11].

It is used to measure the complexity of the molecular structure of organic compounds. Topological indices are designed to quantify the connectivity of atoms in a molecule [12, 13]. The most common topological indices are the Wiener index, Randic index, Szeged index, and Zagreb index [14]. These indices are calculated from the graph of a given molecule, from which the number of vertices, edges, and cycles are determined.

The Wiener index is a measure of the total length of the shortest paths between all pairs of vertices in a molecule. The Randic index is a measure of the number of paths with a given length that join the pair of atoms in a molecule. The Szeged index is a measure of the number of cycles of given length in a molecule. The Zagreb index [15] is a measure of the number of cycles of given length in a molecule.

Topological indices are used in the field of medicinal chemistry to predict the biological activity of compounds. They are also used in the field of drug design to identify compounds that are likely to have the desired biological activity. In addition, topological indices can be used to predict the physical properties of compounds [16], such as boiling point and melting point.

In theoretical chemistry and nanotechnology, there are various graph-related numerical descriptors that are relevant [17–25]. Degree-based descriptors assess a node’s degree, which is the number of edges that connect it. Distance-based descriptors assess the distance between nodes and can be used to determine a node's centrality in the graph. Counting-related graph descriptors count edges, vertices, and other graph constituents. These characteristics are useful for understanding the structure of a graph and comparing different graph architectures [21, 26–33]. The degree-based graph descriptors also be used to identify the number of rings in a molecule and the size of the rings. In addition, they can be used to characterize the topology of a molecule, such as its hydrogen bond network and its connectivity. These graph-based descriptors can also be used to classify molecules into different categories, such as drug-like or non-drug-like. The degree-based graph descriptors provide important insights into the physical and chemical properties of a molecule and can be used to better understand its reactivity and behavior [34]. Researchers are working on connectivity/topological indices in various ways [35, 36]. Some are developing them as graph descriptors, while others are applying them to analyze the chemical properties of molecules [8, 37, 38].

The QSPR and QSAR models are based on the theory of molecular topology, which uses mathematical and statistical techniques to describe the spatial arrangement of atoms and bonds in a molecule [39, 40]. This approach provides insight into the molecular structure, as well as its physical and chemical properties. With the increasing availability of powerful computational methods, these models are becoming increasingly accurate and predictive. By taking into account the topology of a molecule, QSPR and QSAR models can accurately predict the structure–property relationships of a variety of materials and can be used to optimize the design of new materials [10, 41, 42].

Topological indices are numerical descriptors of molecular structure that are derived from the graph theory of a molecule. These indices provide a way to quantify the complexity of the molecular structure and are used to predict a range of molecular properties, such as physical, chemical, and biological activities. Generally, topological indices can be divided into two categories: atom-based and distance-based. Atom-based indices are derived from the connectivity of the atoms in the molecule, while distance-based indices are derived from the distances between the atoms. Both types of indices are used to characterize the overall topology of the molecule, but distance-based indices can provide more information about the shape and size of the molecule.

Topological indices are also used to compare molecules to identify similar structures and to design novel molecules with desirable properties. These indices are also used to identify important structural features for target molecules, such as hydrogen-bonding sites and aromatic rings.

A regular graph is one in which every vertex has the same degree, or number of connections connecting to it. The irregularity topological index of a graph is a graph invariant used to measure how close a graph is to being regular [43].

The irregularity topological indices are important in measuring the complexity of the structure of the molecules. These indices are particularly useful in the design of drug molecules, as they provide a way to measure the complexity of the structure and the ability of the molecule to interact with its target. In addition, these indices are also used to predict the toxicity and other properties of the molecules.

## Preliminaries

We need to first define some fundamentals; let ${\mathcal{H}}$ be a graph with the labels E for the bonds and V for the atoms. Whereas |E| contributed as the number of edges or bonds and |V| represent the total number of nodes or atoms. The irregularity index is a more efficient technique to express irregularity. Recently, a new approach of studying irregularity indices has been developed [44, 45]. The 1st irregularity index was introduced by Bell in 1992 [46]. Most of these indices used the concept of imbalance of an edge defined as ${\text{imball}}_{\mu\nu } = \left| {{\text{d}}_{{_{\mu } }} - {\text{d}}_{\nu } } \right|$.

The Albertson index, AL was defined by Alberston and written as [47]

$${\text{AL}}\left( \mathcal{H} \right) = {\text{~}}\mathop \sum \limits_{{\mu \nu \in {\text{E}}\left( \mathcal{H} \right)}} \left| {{\text{d}}_{\mu } - {\text{d}}_{\nu } } \right|$$

In this index, the imbalance of edges is computed.

The irregularity index IRL and IRLU is introduced by Vukicevic and Gasparov as

$${\text{IRL}}\left( \mathcal{H} \right){\text{~}} = {\text{~}}\mathop \sum \limits_{{\mu \nu \in {\text{E}}\left(\mathcal{H} \right)}} \left| {{\text{lnd}}_{\mu } - {\text{lnd}}_{\nu } } \right|$$

and

$${\text{IRLU}}\left( \mathcal{H} \right) = {\text{~}}\mathop \sum \limits_{{\mu \nu \in {\text{E}}\left( \mathcal{H} \right)}} \frac{{\left| {{\text{d}}_{\mu } - {\text{d}}_{\nu } } \right|}}{{{\text{min}}\left( {{\text{d}}_{\mu } ,{\text{d}}_{\nu } } \right)}}$$

Recently, Abdoo and Dimitrov introduced the new term “total irregularity measure of a graph G”, which is given as [48]

$${\text{IRR}}_{{\text{t}}} \left( \mathcal{H} \right) = {\text{~}}\frac{1}{2}\mathop \sum \limits_{{\mu \nu \in {\text{E}}\left( \mathcal{H} \right)}} \left| {{\text{d}}_{\mu} - {\text{d}}_{\nu } } \right|$$

Recently, Gutman introduced the IRF irregularity index of the graph, which is given as [49]

$${\text{IRF}}\left( \mathcal{H} \right){\text{~}} = {\text{~}}\mathop \sum \limits_{{\mu \nu \in {\text{E}}\left( \mathcal{H} \right)}} \left( {{\text{d}}_{\mu } - {\text{d}}_{\nu } } \right)^{2}$$

The Randic index itself is directly related to an irregularity measure, which is described as [50]

$${\text{IRA}}\left( \mathcal{H} \right) = {\text{~}}\mathop \sum \limits_{{\mu \nu \in {\text{E}}\left( \mathcal{H} \right)}} \left( {{\text{d}}_{\mu }^{{ - 1/2}} - {\text{d}}_{\nu }^{{ - 1/2}} } \right)^{2}$$

The detailed tracing of more irregularity indices of a similar nature is accessible in [15]. These indices are given by

$${\text{IRDIF}}\left( \mathcal{H} \right){\text{~}} = \sum\nolimits_{{\mu \nu \in {\text{E}}\left( \mathcal{H} \right)}} {\left| {\frac{{{\text{d}}_{\mu } }}{{{\text{d}}_{\nu } }} - \frac{{{\text{d}}_{\nu } }}{{{\text{d}}_{\mu } }}} \right|} ,\,{\text{IRLF}}\left( \mathcal{H} \right) = \sum\nolimits_{{\mu \nu \in {\text{E}}\left( \mathcal{H} \right)}} {\frac{{\left| {{\text{d}}_{\mu } - {\text{d}}_{\nu } } \right|}}{{\sqrt {{\text{d}}_{\mu } {\text{d}}_{\nu } } }}} {\text{~}}$$

$${\text{IRLA}}\left( \mathcal{H} \right) = {\text{~}}2\sum\nolimits_{{\mu \nu \in {\text{E}}\left( \mathcal{H} \right)}} {\frac{{\left| {{\text{d}}_{\mu } - {\text{d}}_{\nu } } \right|}}{{\left( {{\text{d}}_{\nu } + {\text{d}}_{\mu } } \right)}}} {\text{~}},\,{\text{IRD}}1\left( \mathcal{H} \right) = {\text{~~}}\sum\nolimits_{{\mu \nu \in {\text{E}}\left( \mathcal{H} \right)}} {{\text{ln}}\left\{ {1 + \left| {{\text{d}}_{\mu } - {\text{d}}_{\nu } } \right|} \right\}}$$

$${\text{IRGA}}\left( \mathcal{H} \right) = {\text{~}}\sum\nolimits_{{\mu \nu \in {\text{E}}\left( \mathcal{H} \right)}} {{\text{ln}}\frac{{\left( {{\text{d}}_{\mu } + {\text{d}}_{\mu } } \right)}}{{2\sqrt {{\text{d}}_{\mu } {\text{d}}_{\nu } } }}} ,\,{\text{IRB}}\left( \mathcal{H} \right){\text{~}} = \sum\nolimits_{{\mu \nu \in {\text{E}}\left( \mathcal{H} \right)}} {\left( {\sqrt {{\text{d}}_{\mu } } - \sqrt {{\text{d}}_{\nu } } } \right)^{2} }$$

Recently, authors computed irregularity indices of a nanotubes [51]. Gao et al. computed irregularity measure of some dendrimer structures and molecular structures [52, 53]. Hussain et al. computed these irregularity measures for some classes of benzenoid systems [54].

## Crystal structure of indium phosphide

**Fig. 1:**
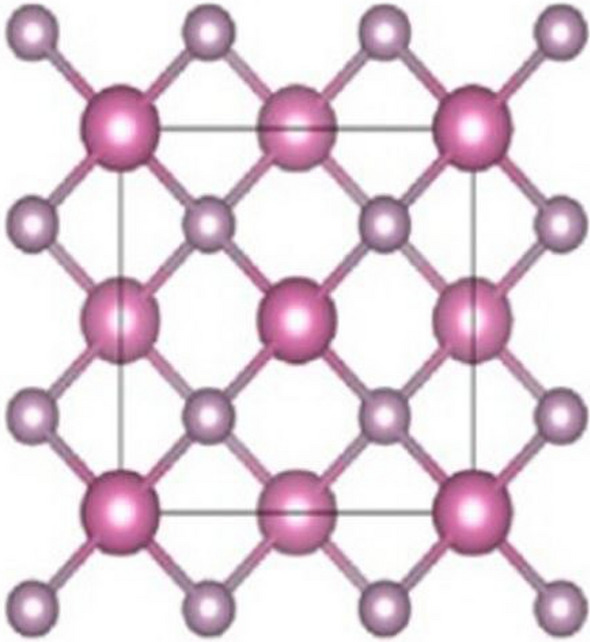
Crystal structure of indium phosphide (InP)

Indium phosphide (InP) is a binary semiconductor composed of indium and phosphorus. It has a zinc blende crystal structure, which is a type of cubic lattice structure. This lattice is composed of indium and phosphorus atoms arranged in a lattice of cube-shaped cells, with each cell containing four indium atoms and four phosphorus atoms. This lattice structure is the same for all materials with a zinc blende crystal structure and is the most common type of lattice structure in semiconductors. Its crystal structure is comparable to that of the majority of group III -V semiconductors, which have a face-centered cubic shape, as illustrated in Fig. 1 [17, 55]. The most common commercial method for synthesizing indium phosphide is known as the Bridgman technique. This method is used to combine refined high temperatures and pressures with indium and phosphorus in a vacuum sealed quartz tube. The tube is placed into a furnace and heated for several hours as the pressure is slowly increased. This process results in a single-crystal ingot of indium phosphide. The results of the electrochemical etching of indium phosphide nano-crystalline surface were studied using scanning electron microscope (SEM). SEM images of the etched surface show that the etching process resulted in the formation of nano-sized pores, with a mean pore size of around 0.2 μm. The etched surface also shows high surface roughness, with the average roughness value of about 2.4 nm. The SEM images also show that the etching process had removed the native oxide layer from the surface.

The number of vertices and edges of ${\text{InP}}[\mathcalligra{r},\,\mathcalligra{s}],$ are $10\mathcalligra{r} \mathcalligra{s} +3\mathcalligra{r} +3\mathcalligra{r} \mathcalligra{s}+2$ and $16\mathcalligra{r} \mathcalligra{s}$ respectively for $\mathcalligra{r}\,\times\,\mathcalligra{s}$ unit cells. Furthermore, Table 1 gives details about the edge partition.

**Table 1:** Edge partition of ${\text{InP}}[\mathcalligra{r},\,\mathcalligra{s}],$ based on degrees of end vertices of each edge

$\left( {{\text{d}}_{\mu } ,{\text{d}}_{\nu } } \right),{\text{~}}\mu \nu \in {\text{InP}}\left[ {\mathcalligra{r} ,\mathcalligra{s} } \right]$	$\left( {{\text{c}}_{\mu } ,{\text{c}}_{\nu } } \right),{\text{~}}\mu \nu \in {\text{InP}}\left[ {\mathcalligra{r} ,\mathcalligra{s} } \right]$	${\text{No}}.\mathrm{of edges}/{\text{frequency}}$
$(\mathrm{1,4})$	$(\mathrm{4,1})$	$4(-1)$
$(\mathrm{2,4})$	$(\mathrm{3,1})$	$4(\partial )$
$(\mathrm{4,4})$	$(\mathrm{1,1})$	$4(2)$

## Main results

In this section we have computed some degree based irregularity topological indices and reverse irregularity indices for ${\text{InP}}[\mathcalligra{r},\,\mathcalligra{s}],$ and derived closed formulas for them, the graph is depicted in Fig. 1. The computational results are as follows:

### Theorem 4.1

*The irregularity indices for the graph of* $\user2{InP}\left[ {\mathcalligra{r},\, \mathcalligra{s} } \right]\user2{~}$ *with *$\mathcalligra{r},\, \mathcalligra{s} {\mathbf{~}} \ge 1$* corresponds to*:

- 1. $${\text{IRDIF}}\left( {{\text{InP}}\left[ {\mathcalligra{r},\, \mathcalligra{s} } \right]} \right) = 6\mathcalligra{r} \mathcalligra{s} + 21\mathcalligra{r} + 21\mathcalligra{s} - 15$$
- 2. $${\text{AL}}\left( {{\text{InP}}\left[ {\mathcalligra{r},\, \mathcalligra{s} } \right]} \right) = 8\mathcalligra{r} \mathcalligra{s} + 20\mathcalligra{r} + 20\mathcalligra{s} - 12$$
- 3. $${\text{IRL}}\left( {{\text{InP}}\left[ {\mathcalligra{r},\, \mathcalligra{s} } \right]} \right) = 2.7724\mathcalligra{r} \mathcalligra{s} + 8.3176\mathcalligra{r} + 8.3176\mathcalligra{s} - 5.5452$$
- 4. $${\text{IRLU}}\left( {{\text{InP}}\left[ {\mathcalligra{r},\, \mathcalligra{s} } \right]} \right) = 4\mathcalligra{r} \mathcalligra{s} + 16\mathcalligra{r} + 16\mathcalligra{s} - 12$$
- 5. $${\text{IRLF}}\left( {{\text{InP}}\left[ {\mathcalligra{r},\, \mathcalligra{s} } \right]} \right) = 2.8284\mathcalligra{r} \mathcalligra{s} + 8.8284\mathcalligra{r} + 8.8284\mathcalligra{s} - 6$$
- 6. $${\text{IRF}}\left( {{\text{InP}}\left[ {\mathcalligra{r},\, \mathcalligra{s} } \right]} \right) = 16\mathcalligra{r} \mathcalligra{s} + 52\mathcalligra{r} + 52\mathcalligra{s} - 36$$
- 7. $${\text{IRLA}}\left( {{\text{InP}}\left[ {\mathcalligra{r},\,\mathcalligra{s} } \right]} \right) = \frac{8}{3}\mathcalligra{r}\mathcalligra{s} + \frac{{112}}{{15}}\mathcalligra{r} + \frac{{112}}{{15}}\mathcalligra{s} - \frac{{24}}{5}$$
- 8. $${\text{IRD1}}\left( {{\text{InP}}\left[ {\mathcalligra{r},\, \mathcalligra{s} } \right]} \right) = 4.3944\mathcalligra{r} \mathcalligra{s} + 9.9396\mathcalligra{r} + 9.9396\mathcalligra{s} - 5.5452$$
- 9. $${\text{IRA}}\left( {{\text{InP}}\left[ {\mathcalligra{r},\, \mathcalligra{s} } \right]} \right) = 0.1716\mathcalligra{r} \mathcalligra{s} + 1.1716\mathcalligra{r} + 1.1716\mathcalligra{s} - 1$$
- 10. $${\text{IRGA}}\left( {{\text{InP}}\left[ {\mathcalligra{r},\, \mathcalligra{s} } \right]} \right) = 0.2356\mathcalligra{r} \mathcalligra{s} + 1.128\mathcalligra{r} + 1.128\mathcalligra{s} - 0.8924$$
- 11. $${\text{IRB}}\left( {{\text{InP}}\left[ {\mathcalligra{r},\, \mathcalligra{s} } \right]} \right) = 1.3724\mathcalligra{r} \mathcalligra{s} + 5.3724\mathcalligra{r} + 5.3724\mathcalligra{s} - 4$$
- 12. $${\text{IRR}}_{{\text{t}}} \left( {{\text{InP}}\left[ {\mathcalligra{r},\,\mathcalligra{s} } \right]} \right) = 4\mathcalligra{r}\mathcalligra{s} + 10\mathcalligra{r} + 10\mathcalligra{s} - 6{\text{~}}$$

### ***Proof***

According to edge partition of ${\text{InP}}[\mathcalligra{r},\,\mathcalligra{s}]$, and above definitions, we computed the irregularity indices, and the computations are given by:

- 1. $${\text{IRDIF}}\left( {{\text{InP}}\left[ {\mathcalligra{r},\,\mathcalligra{s} } \right]} \right){\text{~}} = \sum\nolimits_{{\mu \nu \in {\text{E}}\left( \mathcal{H} \right)}} {\left| {\frac{{{\text{d}}_{\mu } }}{{{\text{d}}_{\nu } }} - \frac{{{\text{d}}_{\nu } }}{{{\text{d}}_{\mu } }}} \right|}$$
  $$=\, \left| {\frac{1}{4} - \frac{4}{1}} \right|\left\{ {4\left( {\mathcalligra{r} + \mathcalligra{s} - 1} \right)} \right\} + {\text{~}}\left| {\frac{2}{4} - \frac{4}{2}} \right|\left\{ {4\left( {\mathcalligra{r}\mathcalligra{s} + \mathcalligra{r} + \mathcalligra{s} } \right)} \right\} + \left| {\frac{4}{4} - \frac{4}{4}} \right|\left\{ {4\left( {2\mathcalligra{r}\mathcalligra{s} - \mathcalligra{r} - \mathcalligra{s} } \right)} \right\}$$
  $$= ~\frac{{15}}{4} \times 4\left( {\mathcalligra{r} + \mathcalligra{s} - 1} \right) + \frac{3}{2} \times 4\left( {\mathcalligra{r}\mathcalligra{s} +\mathcalligra{r} +\mathcalligra{s}} \right) + 0$$
  $$= {\text{~}}15\mathcalligra{r} + 15\mathcalligra{s} - 15 + 6\mathcalligra{r}\mathcalligra{s} + 6\mathcalligra{r} + 6\mathcalligra{s}$$
  $${\text{IRDIF}}\left( {{\text{InP}}\left[ {\mathcalligra{r},\,\mathcalligra{s}} \right]} \right) = {\text{~}}6\mathcalligra{r}\mathcalligra{s} + 21\mathcalligra{r} + 21\mathcalligra{s} - 15$$

- 2. $${\text{AL}}\left( {{\text{InP}}\left[ {\mathcalligra{r} ,\mathcalligra{s} } \right]} \right){\text{~}} = \sum\nolimits_{{\mu \nu \in {\text{E}}\left( \mathcal{H} \right)}} {\left| {d_{\mu } - d_{\nu } } \right|}$$
  $$=\, \left| {1 - 4} \right|\left\{ {4\left( {\mathcalligra{r} + \mathcalligra{s} - 1} \right)} \right\} + {\text{~}}\left| {2 - 4} \right|\left\{ {4\left( {\mathcalligra{r}\mathcalligra{s} + \mathcalligra{r} +\mathcalligra{s} } \right)} \right\} + \left| {4 - 4} \right|\left\{ {4\left( {2\mathcalligra{r}\mathcalligra{s} - \mathcalligra{r} - \mathcalligra{s}} \right)} \right\}$$
  $$= {\text{~}}12\mathcalligra{r} + 12\mathcalligra{s} - 12 + 8\mathcalligra{r}\mathcalligra{s} + 8\mathcalligra{r} + 8\mathcalligra{s}$$
  $${\text{AL}}\left( {{\text{InP}}\left[ {\mathcalligra{r},\,\mathcalligra{s} } \right]} \right) = 8\mathcalligra{r}\mathcalligra{s} + 20\mathcalligra{r} + 20\mathcalligra{s} - 12{\text{~}}$$

- 3. $${\text{IRL}}\left( {{\text{InP}}\left[ {\mathcalligra{r} ,\mathcalligra{s} } \right]} \right){\text{~}} = \sum\nolimits_{{\mu \nu \in {\text{E}}\left( \mathcal{H} \right)}} {\left| {{{\text{lnd}}}_{\mu } - {{\text{lnd}}}_{\nu } } \right|}$$
  $$=\, \left| {{\text{ln}}1 - {\text{ln}}4} \right|\left\{ {4\left( {\mathcalligra{r} + \mathcalligra{s} - 1} \right)} \right\} + \left| {{\text{ln}}2 - {\text{ln}}4} \right|\left\{ {4\left( {\mathcalligra{r}\mathcalligra{s} + \mathcalligra{r} + \mathcalligra{s} } \right)} \right\} + \left| {{\text{ln}}4 - {\text{ln}}4} \right|\left\{ {4\left( {2\mathcalligra{r}\mathcalligra{s} - \mathcalligra{r} - \mathcalligra{s} } \right)} \right\}$$
  $$= ~5.5452 \times \left( {\mathcalligra{r} +\mathcalligra{s} - 1} \right) + 2.7724 \times \left( {\mathcalligra{r}\mathcalligra{s} +\mathcalligra{r} + \mathcalligra{s} } \right) + 0$$
  $${\text{IRL}}\left( {{\text{InP}}\left[ {\mathcalligra{r} ,\mathcalligra{s} } \right]} \right) = 2.7724\mathcalligra{r}\mathcalligra{s} + 8.3176\mathcalligra{r} + 8.3176\mathcalligra{s} - 5.5452{\text{~}}$$

- 4. $${\text{IRLU}}\left( {{\text{InP}}\left[ {\mathcalligra{r} ,\mathcalligra{s} } \right]} \right) = {\text{~}}\sum\nolimits_{{\mu \nu \in {\text{E}}\left( \mathcal{H} \right)}} {\frac{{\left| {{\text{d}}_{\mu } - {\text{d}}_{\nu } } \right|}}{{{\text{min}}\left( {{\text{d}}_{\mu } ,{\text{d}}_{\nu } } \right)}}}$$
  $$= {\text{~}}\frac{{\left| {1 - 4} \right|}}{1}\left\{ {4\left( {\mathcalligra{r} + \mathcalligra{s} - 1} \right)} \right\} + \frac{{\left| {2 - 4} \right|}}{2}\left\{ {4\left( {\mathcalligra{r}\mathcalligra{s} + \mathcalligra{r} + \mathcalligra{s} } \right)} \right\} + \frac{{\left| {4 - 4} \right|}}{4}\left\{ {4\left( {2\mathcalligra{r}\mathcalligra{s} - \mathcalligra{r} - \mathcalligra{s} } \right)} \right\}$$
  $$= ~12\left( {\mathcalligra{r} + \mathcalligra{s} - 1} \right) + 4\left( {\mathcalligra{r}\mathcalligra{s} + \mathcalligra{r} + \mathcalligra{s} } \right) + 0$$
  $${\text{IRLU}}\left( {{\text{InP}}\left[ {\mathcalligra{r} ,\mathcalligra{s} } \right]} \right)\, = \,\,4\mathcalligra{r}\mathcalligra{s} + 16\mathcalligra{r} + 16\mathcalligra{s} - 12$$

- 5. $${\text{IRLF}}\left( {{\text{InP}}\left[ {\mathcalligra{r} ,\mathcalligra{s} } \right]} \right) = \sum\nolimits_{{\mu \nu \in {\text{E}}\left( \mathcal{H} \right)}} {\frac{{\left| {{\text{d}}_{\mu } - {\text{d}}_{\nu } } \right|}}{{\sqrt {{\text{d}}_{\mu } {\text{d}}_{\nu } } }}}$$
  $$= {\text{~}}\frac{{\left| {1 - 4} \right|}}{{\sqrt 4 }}\left\{ {4\left( {\mathcalligra{r} + \mathcalligra{s} - 1} \right)} \right\} + \frac{{\left| {2 - 4} \right|}}{{\sqrt 8 }}\left\{ {4\left( {\mathcalligra{r}\mathcalligra{s} + \mathcalligra{r} + \mathcalligra{s} } \right)} \right\} + \frac{{\left| {4 - 4} \right|}}{{\sqrt {16} }}\left\{ {4\left( {2\mathcalligra{r}\mathcalligra{s} - \mathcalligra{r} - \mathcalligra{s} } \right)} \right\}$$
  $$= ~6\left( {\mathcalligra{r} + \mathcalligra{s} - 1} \right) + 2.8284\left( {\mathcalligra{r}\mathcalligra{s} + \mathcalligra{r} + \mathcalligra{s} } \right) + 0$$
  $${\text{IRLF}}\left( {{\text{InP}}\left[ {\mathcalligra{r} ,\mathcalligra{s} } \right]} \right) = {~}2.8284\mathcalligra{r}\mathcalligra{s} + 8.8284\mathcalligra{r} + 8.8284\mathcalligra{s} - 6$$

- 6. $${\text{IRF}}\left( {{\text{InP}}\left[ {\mathcalligra{r} ,\mathcalligra{s} } \right]} \right){\text{~}} = \sum\nolimits_{{\mu \nu \in {\text{E}}\left( \mathcal{H} \right)}} {\left( {{\text{d}}_{\mu } - {\text{d}}_{\nu } } \right)^{2} }$$
  $$= \left( {1 - 4} \right)^{2} \left\{ {4\left( {\mathcalligra{r} + \mathcalligra{s} - 1} \right)} \right\} + \left( {2 - 4} \right)^{2} \left\{ {4\left( {\mathcalligra{r}\mathcalligra{s} + \mathcalligra{r} + \mathcalligra{s}} \right)} \right\} + \left( {4 - 4} \right)^{2} \left\{ {4\left( {2\mathcalligra{r}\mathcalligra{s} - \mathcalligra{r} - \mathcalligra{r} } \right)} \right\}$$
  $$= ~36\left( {\mathcalligra{r} +\mathcalligra{r} - 1} \right) + 16\left( {\mathcalligra{r}\mathcalligra{s} + \mathcalligra{r} + \mathcalligra{s} } \right) + 0$$
  $${\text{IRF}}\left( {{\text{InP}}\left[ {\mathcalligra{r} ,\mathcalligra{s} } \right]} \right) = {~}16\mathcalligra{r}\mathcalligra{s} + 52\mathcalligra{r} + 52\mathcalligra{s} - 36$$

- 7. $${\text{IRLA}}\left( {{\text{InP}}\left[ {\mathcalligra{r} ,\mathcalligra{s} } \right]} \right) = {\text{~}}2{\text{~}}\sum\nolimits_{{\mu \nu \in {\text{E}}\left( \mathcal{H} \right)}} {\frac{{\left| {{\text{d}}_{\mu } - {\text{d}}_{\nu } } \right.}}{{\left( {{\text{d}}_{\mu } + {\text{d}}_{\nu } } \right)}}}$$
  $$=\, 2\left[ {\frac{{\left| {1 - 4} \right|}}{{\left( {1 + 4} \right)}}\left\{ {4\left( {\mathcalligra{r} + \mathcalligra{s} - 1} \right)} \right\}\, + \,\frac{{\left| {2 - 4} \right|}}{{\left( {2 + 4} \right)}}\left\{ {4\left( {\mathcalligra{r}\mathcalligra{s} + \mathcalligra{r} + \mathcalligra{s} } \right)} \right\} + \frac{{\left| {4 - 4} \right|}}{{\left( {4 + 4} \right)}}\left\{ {4\left( {2\mathcalligra{r}\mathcalligra{s} - \mathcalligra{r} - \mathcalligra{s} } \right)} \right.} \right]$$
  $$= 2\left[~\frac{{12}}{5}\left( {\mathcalligra{r} +\mathcalligra{s} - 1} \right) + \frac{4}{3}\left( {\mathcalligra{r}\mathcalligra{s} + \mathcalligra{r} + \mathcalligra{s} } \right) + 0 \right]$$
  $$= ~\frac{{24}}{5}\left( {\mathcalligra{r} + \mathcalligra{s} - 1} \right) + \frac{8}{3}\left( {\mathcalligra{r}\mathcalligra{s} + \mathcalligra{r} +\mathcalligra{s} } \right)$$
  $${\text{IRLA}}\left( {{\text{InP}}\left[ {\mathcalligra{r} ,\mathcalligra{s} } \right]} \right) = \frac{8}{3}\mathcalligra{r}\mathcalligra{s} + \frac{{112}}{{15}}\mathcalligra{r} + \frac{{112}}{{15}}\mathcalligra{r} - \frac{{24}}{5}$$

- 8. $${\text{IRD}}1\left( {{\text{InP}}\left[ {\mathcalligra{r} ,\mathcalligra{r} } \right]} \right) = \sum\nolimits_{{\mu \nu \in {\text{E}}\left( \mathcal{H} \right)}} {{\text{ln}}\left\{ {1 + \left| {{\text{d}}_{\mu } - {\text{d}}_{\nu } } \right|} \right\}}$$
  $$= \ln \left\{ {1 + \left| {1 - 4} \right|{\text{~}}} \right\}\left\{ {4\left( {\mathcalligra{r} + \mathcalligra{r} - 1} \right)} \right\} + \ln \left\{ {1 + \left| {2 - 4} \right|{\text{~}}} \right\}\left\{ {4\left( {\mathcalligra{r} \mathcalligra{s} + \mathcalligra{r} + \mathcalligra{s} } \right)} \right\} + {\text{ln}}\left\{ {1 + \left| {4 - 4} \right|} \right\}\left\{ {4\left( {2\mathcalligra{r}\mathcalligra{s} - \mathcalligra{r} - \mathcalligra{s} } \right)} \right\}$$
  $$= \ln 4 \times \left\{ {4\left( {\mathcalligra{r} + \mathcalligra{s} - 1} \right)} \right\} + \ln 3 \times \left\{ {4\left( {\mathcalligra{r} \mathcalligra{s} + \mathcalligra{r} + \mathcalligra{s} } \right)} \right\} + 0$$
  $$= 5.5452\left( {\mathcalligra{r} + \mathcalligra{s} - 1} \right) + 4.3944\left( {\mathcalligra{r}\mathcalligra{s} + \mathcalligra{r} + \mathcalligra{s} } \right)$$
  $${\text{IRD}}1\left( {{\text{InP}}\left[ {\mathcalligra{r} ,\mathcalligra{s} } \right]} \right) = 4.3944\mathcalligra{r}\mathcalligra{s} + 9.9396\mathcalligra{r} + 9.9396\mathcalligra{s} - 5.5452$$

- 9. $${\text{IRA}}\left( {{\text{InP}}\left[ {\mathcalligra{r} ,\mathcalligra{s} } \right]} \right) = \sum\nolimits_{{\mu \nu \in {\text{E}}\left( \mathcal{H} \right)}} {\left( {{\text{d}}_{\mu }^{{ - 1/2}} - {\text{d}}_{\nu }^{{ - 1/2}} } \right)^{2} } {\text{~}}$$
  $$= \left( {1^{{ - 1/2}} - 4^{{ - 1/2}} } \right)^{2} \left\{ {4\left( {\mathcalligra{r} + \mathcalligra{s} - 1} \right)} \right\} + \left( {2^{{ - 1/2}} - 4^{{ - 1/2}} } \right)^{2} \left\{ {4\left( {\mathcalligra{r}\mathcalligra{s} +\mathcalligra{r} + \mathcalligra{s} } \right)} \right\} + \left( {4^{{ - 1/2}} - 4^{{ - 1/2}} } \right)^{2} \left\{ {4\left( {2\mathcalligra{r}\mathcalligra{s} - \mathcalligra{r} - \mathcalligra{s} } \right)} \right\}$$
  $$= {\text{~}}\left( {\mathcalligra{r} + \mathcalligra{s} - 1} \right) + 0.1716\left( {\mathcalligra{r}\mathcalligra{s} + \mathcalligra{r} + \mathcalligra{s} } \right)$$
  $${\text{IRA}}\left( {{\text{InP}}\left[ {\mathcalligra{r} ,\mathcalligra{s} } \right]} \right) = {\text{~}}0.1716\mathcalligra{r}\mathcalligra{s} + 1.1716\mathcalligra{r} + 1.1716\mathcalligra{s} - 1$$

- 10. $${\text{IRGA}}\left( {{\text{InP}}\left[ {\mathcalligra{r} ,\mathcalligra{r}} \right]} \right) = {\text{~}}\sum\nolimits_{{\mu \nu \in {\text{E}}\left( \mathcal{H} \right)}} {{\text{ln}}\frac{{\left( {{\text{d}}_{\mu } + {\text{d}}_{\nu } } \right)}}{{2\sqrt {{\text{d}}_{\mu } {\text{d}}_{\nu } } }}}$$
  $$= {\text{~ln}}\frac{{\left( {1 + 4} \right)}}{{2\sqrt 4 }}\left\{ {4\left( {\mathcalligra{r} + \mathcalligra{r} - 1} \right)} \right\} + {\text{ln}}\frac{{\left( {2 + 4} \right)}}{{2\sqrt 8 }}\left\{ {4\left( {\mathcalligra{r}\mathcalligra{s} + \mathcalligra{r} + \mathcalligra{r} } \right)} \right\} + {\text{ln}}\frac{{\left( {4 + 4} \right)}}{{2\sqrt {16} }}\left\{ {4\left( {2\mathcalligra{r}\mathcalligra{s} - \mathcalligra{r} - \mathcalligra{s} } \right)} \right\}$$
  $$= ~0.2231 \times 4\left( {\mathcalligra{r} + \mathcalligra{s} - 1} \right) + 0.0589 \times 4\left( {\mathcalligra{r}\mathcalligra{s} + \mathcalligra{r} + \mathcalligra{s} } \right) + 0$$
  $$= ~0.8924\left( {\mathcalligra{r} + \mathcalligra{s} - 1} \right) + 0.2356\left( {\mathcalligra{r}\mathcalligra{s} + \mathcalligra{r} + \mathcalligra{s} } \right)$$
  $${\text{IRGA}}\left( {{\text{InP}}\left[ {\mathcalligra{r} ,\mathcalligra{s} } \right]} \right) = 0.2356\mathcalligra{r}\mathcalligra{s} + 1.128\mathcalligra{r} + 1.128\mathcalligra{s} - 0.8924$$

- 11. $${\text{IRB}}\left( {{\text{InP}}\left[ {\mathcalligra{r} ,\mathcalligra{s} } \right]} \right){\text{~}} = \mathop \sum \limits_{{\mu \nu \in {\text{E}}\left( \mathcal{H} \right)}} \left( {\sqrt {{\text{d}}_{\mu } } - \sqrt {{\text{d}}_{\nu } } } \right)^{2}$$
  $$= {\text{~}}\left( {\sqrt 1 - \sqrt 4 } \right)^{2} \left\{ {4\left( {\mathcalligra{r} + \mathcalligra{s} - 1} \right)} \right\} + \left( {\sqrt 2 - \sqrt 4 } \right)^{2} \left\{ {4\left( {\mathcalligra{r}\mathcalligra{s} + \mathcalligra{r} + \mathcalligra{s} } \right)} \right\} + \left( {\sqrt 4 - \sqrt 4 } \right)^{2} \left\{ {4\left( {2\mathcalligra{r}\mathcalligra{s} - \mathcalligra{r} - \mathcalligra{s} } \right)} \right\}$$
  $$= 4\left( {\mathcalligra{r} + \mathcalligra{s} - 1} \right) + 1.3724\left( {\mathcalligra{r}\mathcalligra{s} + \mathcalligra{r} + \mathcalligra{s} } \right)$$
  $${\text{IRB}}\left( {{\text{InP}}\left[ {\mathcalligra{r} ,\mathcalligra{s} } \right]} \right) = 1.3724\mathcalligra{r}\mathcalligra{s} + 5.3724\mathcalligra{r} + 5.3724\mathcalligra{s} - 4$$

- 12. $$= \frac{1}{2}\left[ {\left| {1 - 4} \right|\left\{ {4\left( {\mathcalligra{r} + \mathcalligra{s} - 1} \right)} \right\} + {\text{~}}\left| {2 - 4} \right|\left\{ {4\left( {\mathcalligra{r}\mathcalligra{s} + \mathcalligra{r} + \mathcalligra{s} } \right)} \right\} + \left| {4 - 4} \right|\left\{ {4\left( {2\mathcalligra{r}\mathcalligra{s} - \mathcalligra{r} - \mathcalligra{s} } \right)} \right\}} \right]$$
  $$= \frac{1}{2}\left[ {\left| {1 - 4} \right|\left\{ {4\left( {\mathcalligra{r} + \mathcalligra{s} - 1} \right)} \right\} + \left| {2 - 4} \right|\left\{ {4\left( {\mathcalligra{r}\mathcalligra{s} + \mathcalligra{r} + \mathcalligra{s} } \right)} \right\} + \left| {4 - 4} \right|\left\{ {4\left( {2\mathcalligra{r}\mathcalligra{s} - \mathcalligra{r} - \mathcalligra{s} } \right)} \right\}} \right]$$
  $$= ~6\left( {\mathcalligra{r} + \mathcalligra{s} - 1} \right) + 4\left( {\mathcalligra{r}\mathcalligra{s} + \mathcalligra{r} + \mathcalligra{s} } \right)$$
  $${\text{IRR}}_{{\text{t}}} \left( {{\text{InP}}\left[ {\mathcalligra{r} ,\mathcalligra{s} } \right]} \right) = 4\mathcalligra{r}\mathcalligra{s} + 10\mathcalligra{r} + 10\mathcalligra{s} - 6{\text{~}}$$

### Theorem 4.2

*The reverse irregularity indices for the graph of* ${\mathbf{InP}} \left[\mathcalligra{r},{\mathbf{\mathcalligra{s}}} \right]$ *with* $\mathcalligra{r} ,{\mathbf{\mathcalligra{s}}} {~} \ge {\mathbf{1}}$ *are corresponding to*:

- 1. $${\mathbf{CIRDIF}}\left( {{\mathbf{InP}}\left[ {\mathcalligra{r} ,{\mathbf{\mathcalligra{r} }}} \right]} \right) = \frac{{32}}{3}\mathcalligra{r}\mathcalligra{s} + \frac{{77}}{3}\mathcalligra{r} + \frac{{77}}{3}\mathcalligra{s} - {\mathbf{15}}$$
- 2. $${\text{CAL}}\left( {{\text{InP}}\left[ {\mathcalligra{r} ,\mathcalligra{s} } \right]} \right) = 8\mathcalligra{r}\mathcalligra{s} + 20\mathcalligra{r} + 20\mathcalligra{s} - 12$$
- 3. $${\text{CIRL}}\left( {{\text{InP}}\left[ {\mathcalligra{r} ,\mathcalligra{s} } \right]} \right) = 4.3944\mathcalligra{r}\mathcalligra{s} + 9.9396\mathcalligra{r} + 9.9396\mathcalligra{s} - 5.5452$$
- 4. $${\text{CIRLU}}\left( {{\text{InP}}\left[ {\mathcalligra{r} ,\mathcalligra{s} } \right]} \right) = 8\mathcalligra{r}\mathcalligra{s} + 20\mathcalligra{r} + 20\mathcalligra{s} - 12$$
- 5. $${\text{CIRLF}}\left( {{\text{InP}}\left[ {\mathcalligra{r} ,\mathcalligra{s} } \right]} \right) = 4.6188\mathcalligra{r}\mathcalligra{s} + 10.6188\mathcalligra{r} + 10.6188\mathcalligra{s} - 6$$
- 6. $${\text{CIRF}}\left( {{\text{InP}}\left[ {\mathcalligra{r} ,\mathcalligra{s} } \right]} \right) = 16\mathcalligra{r}\mathcalligra{s} + 52\mathcalligra{r} + 52\mathcalligra{s} - 36$$
- 7. $${\text{CIRLA}}\left( {{\text{InP}}\left[ {\mathcalligra{r} ,\mathcalligra{s} } \right]} \right) = 4\mathcalligra{r}\mathcalligra{s} + \frac{{44}}{5}\mathcalligra{r} + \frac{{44}}{5}\mathcalligra{s} - \frac{{24}}{5}$$
- 8. $${\text{CIRD1}}\left( {{\text{InP}}\left[ {\mathcalligra{r} ,\mathcalligra{s} } \right]} \right) = 4.3944\mathcalligra{r}\mathcalligra{s} + 9.9396\mathcalligra{r} + 9.9396\mathcalligra{s} - 5.5452$$
- 9. $${\text{CIRA}}\left( {{\text{InP}}\left[ {\mathcalligra{r} ,\mathcalligra{s} } \right]} \right) = 0.7144\mathcalligra{r}\mathcalligra{s} + 1.7144\mathcalligra{r} + 1.7144\mathcalligra{s} - 1$$
- 10. $${\text{CIRGA}}\left( {{\text{InP}}\left[ {\mathcalligra{r} ,\mathcalligra{s} } \right]} \right) = 0.5752\mathcalligra{r}\mathcalligra{s} + 1.4676\mathcalligra{r} + 1.4676\mathcalligra{s} - 0.8924$$
- 11. $${\text{CIRB}}\left( {{\text{InP}}\left[ {\mathcalligra{r} ,\mathcalligra{s} } \right]} \right) = 2.1436\mathcalligra{r}\mathcalligra{s} + 6.1436\mathcalligra{r} + 6.1436\mathcalligra{s} - 4$$
- 12. $${\text{CIRR}}_{{\text{t}}} \left( {{\text{InP}}\left[ {\mathcalligra{r} ,\mathcalligra{s} } \right]} \right) = 4\mathcalligra{r}\mathcalligra{s} + 10\mathcalligra{r} + 10\mathcalligra{s} - 6$$

### ***Proof***

According to edge partition of ${\text{InP}}[\mathcalligra{r},\mathcalligra{s} ]$, and above definitions, we computed the irregularity indices, and the computations are given by:

- 1. $${\text{CIRDIF}}\left( {{\text{InP}}\left[ {\mathcalligra{r} ,\mathcalligra{s} } \right]} \right){\text{~}} = \sum\nolimits_{{\mu \nu \in {\text{E}}\left( \mathcal{H} \right)}} {\left| {\frac{{{\text{c}}_{\mu } }}{{{\text{c}}_{\nu } }} - \frac{{{\text{c}}_{\nu } }}{{{\text{c}}_{\mu } }}} \right|}$$
  $$=\, \left| {\frac{4}{1} - \frac{1}{4}} \right|\left\{ {4\left( {\mathcalligra{r} + \mathcalligra{s} - 1} \right)} \right\} + {\text{~}}\left| {\frac{3}{1} - \frac{1}{3}} \right|\left\{ {4\left( {\mathcalligra{r}\mathcalligra{s} + \mathcalligra{r} + \mathcalligra{s} } \right)} \right\} + \left| {\frac{1}{1} - \frac{1}{1}} \right|\left\{ {4\left( {2\mathcalligra{r}\mathcalligra{s} - \mathcalligra{r} - \mathcalligra{s} } \right)} \right\}$$
  $$= ~15 \times \left( {\mathcalligra{r} + \mathcalligra{s} - 1} \right) + \frac{{32}}{3}\left( {\mathcalligra{r}\mathcalligra{s} + \mathcalligra{r} + \mathcalligra{s} } \right) + 0$$
  $$= {\text{~}}15\mathcalligra{r} + 15\mathcalligra{s} - 15 + \frac{{32}}{3}\mathcalligra{r}\mathcalligra{s} + \frac{{32}}{3}\mathcalligra{r} + \frac{{32}}{3}\mathcalligra{s}$$
  $${\text{CIRDIF}}\left( {{\text{InP}}\left[ {\mathcalligra{r} ,\mathcalligra{s} } \right]} \right) = \frac{{32}}{3}\mathcalligra{r}\mathcalligra{s} + \frac{{77}}{3}\mathcalligra{r} + \frac{{77}}{3}\mathcalligra{s} - 15$$

- 2. $${\text{CAL}}\left( {{\text{InP}}\left[ {\mathcalligra{r} ,\mathcalligra{s} } \right]} \right) = \sum\nolimits_{{\mu \nu \in {\text{E}}\left( \mathcal{H} \right)}} {\left| {{\text{c}}_{\mu } - {\text{c}}_{\nu } } \right|} {\text{~}}$$
  $$= \left| {4 - 1} \right|\left\{ {4\left( {\mathcalligra{r} + \mathcalligra{s} - 1} \right)} \right\} + \left| {3 - 1} \right|\left\{ {4\left( {\mathcalligra{r}\mathcalligra{s} + \mathcalligra{r} + \mathcalligra{s} } \right)} \right\} + \left| {1 - 1} \right|\left\{ {4\left( {2\mathcalligra{r}\mathcalligra{s} - \mathcalligra{r} - \mathcalligra{s} } \right)} \right\}$$
  $$= {\text{~}}12\mathcalligra{r} + 12\mathcalligra{r} - 12 + 8\mathcalligra{r}\mathcalligra{s} + 8\mathcalligra{r} + 8\mathcalligra{s}$$
  $${\text{CAL}}\left( {{\text{InP}}\left[ {\mathcalligra{r} ,\mathcalligra{s} } \right]} \right) = 8\mathcalligra{r}\mathcalligra{s} + 20\mathcalligra{r} + 20\mathcalligra{s} - 12{\text{~}}$$

- 3. $${\text{CIRL}}\left( {{\text{InP}}\left[ {\mathcalligra{r} ,\mathcalligra{s} } \right]} \right) = \sum\nolimits_{{\mu \nu \in {\text{E}}\left( \mathcal{H} \right)}} {\left| {{\text{lnc}}_{\mu } - {\text{lnc}}_{\nu } } \right|}$$
  $$=\, \left| {{\text{ln}}4 - {\text{ln}}1} \right|\left\{ {4\left( {\mathcalligra{r} + \mathcalligra{s} - 1} \right)} \right\} + \left| {{\text{ln}}3 - {\text{ln}}1} \right|\left\{ {4\left( {\mathcalligra{r}\mathcalligra{s} + \mathcalligra{r} + \mathcalligra{s} } \right)} \right\} + \left| {{\text{ln}}1 - {\text{ln}}1} \right|\left\{ {4\left( {2\mathcalligra{r}\mathcalligra{s} - \mathcalligra{r} - \mathcalligra{s} } \right)} \right\}$$
  $$= ~5.5452 \times \left( {\mathcalligra{r} +\mathcalligra{s} - 1} \right) + 4.3944 \times \left( {\mathcalligra{r}\mathcalligra{s} + \mathcalligra{r} + \mathcalligra{s} } \right) + 0$$
  $${\text{CIRL}}\left( {{\text{InP}}\left[ {\mathcalligra{r} ,\mathcalligra{s} } \right]} \right) = 4.3944\mathcalligra{r}\mathcalligra{s} + 9.9396\mathcalligra{r} + 9.9396\mathcalligra{s} - 5.5452{\text{~}}$$

- 4. $${\text{CIRLU}}\left( {{\text{InP}}\left[ {\mathcalligra{r} ,\mathcalligra{s} } \right]} \right) = \sum\nolimits_{{\mu \nu \in {\text{E}}\left( \mathcal{H} \right)}} {\frac{{\left| {{\text{c}}_{\mu } - {\text{c}}_{\nu } } \right|}}{{{\text{min}}\left( {{\text{c}}_{\mu } ,{\text{c}}_{\nu } } \right)}}}$$
  $$= {\text{~}}\frac{{\left| {4 - 1} \right|}}{1}\left\{ {4\left( {\mathcalligra{r} + \mathcalligra{s} - 1} \right)} \right\} + \frac{{\left| {3 - 1} \right|}}{1}\left\{ {4\left( {\mathcalligra{r}\mathcalligra{s} + \mathcalligra{r} + \mathcalligra{s} } \right)} \right\} + \frac{{\left| {1 - 1} \right|}}{1}\left\{ {4\left( {2\mathcalligra{r}\mathcalligra{s} - \mathcalligra{r} - \mathcalligra{s} } \right)} \right\}$$
  $$= ~12\left( {\mathcalligra{r} + \mathcalligra{s} - 1} \right) + 8\left( {\mathcalligra{r}\mathcalligra{s} + \mathcalligra{r} + \mathcalligra{s} } \right) + 0$$
  $${\text{CIRLU}}\left( {{\text{InP}}\left[ {\mathcalligra{r} ,\mathcalligra{s} } \right]} \right) = {~}8\mathcalligra{r} \mathcalligra{s} + 20\mathcalligra{r} + 20\mathcalligra{s} - 12$$

- 5. $${\text{CIRLF}}\left( {{\text{InP}}\left[ {\mathcalligra{r} ,\mathcalligra{r} } \right]} \right) = \sum\nolimits_{{\mu \nu \in {\text{E}}\left( \mathcal{H} \right)}} {\frac{{\left| {{\text{c}}_{\mu } - {\text{c}}_{\nu } } \right|}}{{\sqrt {{\text{c}}_{\mu } {\text{c}}_{\nu } } }}} {\text{~}}$$
  $$= {\text{~}}\frac{{\left| {4 - 1} \right|}}{{\sqrt 4 }}\left\{ {4\left( {\mathcalligra{r} + \mathcalligra{s} - 1} \right)} \right\} + \frac{{\left| {3 - 1} \right|}}{{\sqrt 3 }}\left\{ {4\left( {\mathcalligra{r}\mathcalligra{s} + \mathcalligra{r} + \mathcalligra{s} } \right)} \right\} + \frac{{\left| {1 - 1} \right|}}{{\sqrt 1 }}\left\{ {4\left( {2\mathcalligra{r}\mathcalligra{s} - \mathcalligra{r} - \mathcalligra{s} } \right)} \right\}$$
  $$= ~6\left( {\mathcalligra{r} + \mathcalligra{s} - 1} \right) + 4.6188\left( {\mathcalligra{r}\mathcalligra{s} + \mathcalligra{r} + \mathcalligra{s} } \right) + 0$$
  $${\text{CIRLF}}\left( {{\text{InP}}\left[ {\mathcalligra{r} ,\mathcalligra{s} } \right]} \right) = {~}4.6188\mathcalligra{r}\mathcalligra{s} + 10.6188\mathcalligra{r} + 10.6188\mathcalligra{s} - 6$$

- 6. $${\text{CIRF}}\left( {{\text{InP}}\left[ {\mathcalligra{r} ,\mathcalligra{s} } \right]} \right){\text{~}} = {\text{~}}\sum\nolimits_{{\mu \nu \in {\text{E}}\left( \mathcal{H} \right)}} {\left( {{\text{c}}_{\mu } - {\text{c}}_{\nu } } \right)^{2} }$$
  $$= \,\left( {4 - 1} \right)^{2} \left\{ {4\left( {\mathcalligra{r} + \mathcalligra{s} - 1} \right)} \right\} + \left( {3 - 1} \right)^{2} \left\{ {4\left( {\mathcalligra{r}\mathcalligra{s} + \mathcalligra{r} + \mathcalligra{s} } \right)} \right\} + \left( {1 - 1} \right)^{2} \left\{ {4\left( {2\mathcalligra{r}\mathcalligra{s} - \mathcalligra{r} - \mathcalligra{s} } \right)} \right\}$$
  $$= ~36\left( {\mathcalligra{r} + \mathcalligra{s} - 1} \right) + 16\left( {\mathcalligra{r}\mathcalligra{s} + \mathcalligra{r} + \mathcalligra{s} } \right) + 0$$
  $${\text{CIRF}}\left( {{\text{InP}}\left[ {\mathcalligra{r} ,\mathcalligra{r} } \right]} \right) = {~}16\mathcalligra{r}\mathcalligra{s} + 52\mathcalligra{r} + 52\mathcalligra{s} - 36$$

- 7. $${\text{CIRLA}}\left( {{\text{InP}}\left[ {\mathcalligra{r} ,\mathcalligra{s} } \right]} \right) = {\text{~}}2\sum\nolimits_{{\mu \nu \in {\text{E}}\left( \mathcal{H} \right)}} {\frac{{\left| {{\text{c}}_{\mu } - {\text{c}}_{\nu } } \right|}}{{\left( {{\text{c}}_{\mu } + {\text{c}}_{\nu } } \right)}}} {\text{~}}$$
  $$=\, 2\left[ {\frac{{\left| {4 - 1} \right|}}{{\left( {1 + 4} \right)}}\left\{ {4\left( {\mathcalligra{r} + \mathcalligra{s} - 1} \right)} \right\} + \frac{{\left| {3 - 1} \right|}}{{\left( {3 + 1} \right)}}\left\{ {4\left( {\mathcalligra{r}\mathcalligra{s} + \mathcalligra{r} + \mathcalligra{s} } \right)} \right\} + \frac{{\left| {1 - 1} \right|}}{{\left( {1 + 1} \right)}}\{ 4\left( {2\mathcalligra{r}\mathcalligra{s} - \mathcalligra{r} - \mathcalligra{s} } \right)} \right]$$
  $$= 2\left[ {\frac{{12}}{5}\left( {\mathcalligra{r} + \mathcalligra{s} - 1} \right) + \frac{8}{4}\left( {\mathcalligra{r}\mathcalligra{s} + \mathcalligra{r} + \mathcalligra{s} } \right) + 0} \right]$$
  $$= ~\frac{{24}}{5}\left( {\mathcalligra{r} + \mathcalligra{s} - 1} \right) + 4\left( {\mathcalligra{r}\mathcalligra{s} + \mathcalligra{r} + \mathcalligra{s} } \right)$$
  $${\text{CIRLA}}\left( {{\text{InP}}\left[ {\mathcalligra{r} ,\mathcalligra{s} } \right]} \right) = 4\mathcalligra{r}\mathcalligra{s} + \frac{{44}}{5}\mathcalligra{r} + \frac{{44}}{5}\mathcalligra{s} - \frac{{24}}{5}$$

- 8. $${\text{CIRD}}1\left( {{\text{InP}}\left[ {\mathcalligra{r} ,\mathcalligra{s} } \right]} \right) = {\text{~~}}\mathop \sum \limits_{{\mu \nu \in {\text{E}}\left( \mathcal{H} \right)}} {\text{ln}}\left\{ {1\, + \,\left| {{\text{c}}_{\mu } - {\text{c}}_{\nu } } \right|} \right\}$$

$$=\,{\text{ln}}\left\{1+|4-1| \right\}\{4\left(\mathcalligra{r}+\mathcalligra{s}-1\right)\}+{\text{ln}}\left\{1+|3-1| \right\}\{4(\mathcalligra{r}\mathcalligra{s}+\mathcalligra{r}+\mathcalligra{s})\}+{\text{ln}}\{1+|1-1| \}\{4(2\mathcalligra{r}\mathcalligra{s}-\mathcalligra{r}-\mathcalligra{s})\}$$

$$={\text{ln}}4\times \{4\left(\mathcalligra{r}+\mathcalligra{s}-1\right)\}+{\text{ln}}3\times \left\{4\left(\mathcalligra{r}\mathcalligra{s}+\mathcalligra{r}+\mathcalligra{s} \right)\right\}+0$$

$$=5.5452\left(\mathcalligra{r}+\mathcalligra{s}-1\right)+4.3944(\mathcalligra{r}\mathcalligra{s}+\mathcalligra{r}+\mathcalligra{s})$$

$${\text{CIRD}}1({\text{InP}}[\mathcalligra{r},\,\mathcalligra{s}])=4.3944\mathcalligra{r}\mathcalligra{s}+9.9396\mathcalligra{r}+9.9396\mathcalligra{s}-5.5452$$

- 9. $${\text{CIRA}}\left({\text{InP}}[\mathcalligra{r},\,\mathcalligra{r}]\right)= \sum_{\mu \in {\text{E}}\left(\mu \right)}{\left({{\text{c}}}_{\mu }^{-1/2}-{{\text{c}}}_{\nu }^{-1/2}\right)}^{2}$$
  $$={{\left({4}_{ }^{-1/2}-{1}_{ }^{-1/2}\right)}^{2}\{4\left(\mathcalligra{r}+\mathcalligra{s}-1\right)\}}^{ }+{\left({3}_{ }^{-1/2}-1\right)}^{2}\{4(\mathcalligra{r}\mathcalligra{s}+\mathcalligra{r}+\mathcalligra{s})\}+{\left({1}_{ }^{-1/2}-{1}_{ }^{-1/2}\right)}^{2}\{4(2\mathcalligra{r}\mathcalligra{s}-\mathcalligra{r}-\mathcalligra{s})\}$$
  $$= \left(\mathcalligra{r}+\mathcalligra{s}-1\right)+0.7144(\mathcalligra{r}\mathcalligra{s}+\mathcalligra{r}+\mathcalligra{s})$$
  $${\text{CIRA}}\left({\text{InP}}[\mathcalligra{r},\,\mathcalligra{s}]\right)= 0.7144\mathcalligra{r}\mathcalligra{s}+1.7144\mathcalligra{r}+1.7144\mathcalligra{s}-1$$

- 10. $${\text{CIRGA}}\left({\text{InP}}[\mathcalligra{r}, \mathcalligra{r}]\right)= \sum_{\mu\nu \in {\text{E}}\left(\mu \right)}{\text{ln}}\frac{({{\text{c}}}_{\mu }+{{\text{c}}}_{|nu})}{2\sqrt{{{\text{c}}}_{\mu }\nu }}$$
  $$=\,\mathrm{ ln}\frac{(4+1)}{2\sqrt{4}}\{4\left(\mathcalligra{r}+\mathcalligra{s}-1\right)\}+{\text{ln}}\frac{\left(3+1\right)}{2\sqrt{3}}\{4(\mathcalligra{r}\mathcalligra{s}+\mathcalligra{r}+\mathcalligra{s})\}+{\text{ln}}\frac{\left(1+1\right)}{2\sqrt{1}}\{4(2\mathcalligra{r}\mathcalligra{s}-\mathcalligra{r}-\mathcalligra{s})\}$$
  $$= 0.2231\times 4\left(\mathcalligra{r}+\mathcalligra{s}-1\right)+0.1438\times 4\left(\mathcalligra{r}\mathcalligra{s}+\mathcalligra{r}+\mathcalligra{s} \right)+0$$
  $$= 0.8924\left(\mathcalligra{r}+\mathcalligra{s}-1\right)+0.5752\left(\mathcalligra{r}\mathcalligra{s}+\mathcalligra{r}+\mathcalligra{s} \right)$$
  $${\text{CIRGA}}\left({\text{InP}}[\mathcalligra{r},\,\mathcalligra{s}\right)=0.5752\mathcalligra{r}\mathcalligra{s}+1.4676\mathcalligra{r}+1.4676\mathcalligra{s}-0.8924$$

- 11. $${\text{CIRB}}\left({\text{InP}}[\mathcalligra{r},\,\mathcalligra{s}]\right) =\sum_{\mu\nu \in {\text{E}}\left(\mu \right)}{(\sqrt{{{\text{c}}}_{\mu }}-\sqrt{{{\text{c}}}_{\nu}})}^{2}$$
  $$= {\left(\sqrt{4}-\sqrt{1}\right)}^{2}\{4\left(\mathcalligra{r} + \mathcalligra{s}-1\right)\}+{\left(\sqrt{3}-\sqrt{1}\right)}^{2}\{4(\mathcalligra{r}\mathcalligra{s}) + \mathcalligra{r} + \mathcalligra{s}\}+{\left(\sqrt{1}-\sqrt{1}\right)}^{2}\{4(2\mathcalligra{r}\mathcalligra{s} - \mathcalligra{r} - \mathcalligra{s})\}$$
  $$=4\left(\mathcalligra{r}+\mathcalligra{s}-1\right)+2.1436(\mathcalligra{r}\mathcalligra{s} + \mathcalligra{r} + \mathcalligra{s})$$
  $${\text{CIRB}}\left({\text{InP}}[\mathcalligra{r}], \mathcalligra{s}\right)=2.1436\mathcalligra{r}\mathcalligra{s}+6.1436\mathcalligra{r}+6.1436\mathcalligra{s}-4$$

- 12. $${{\text{CIRR}}}_{{\text{t}}}\left({\text{InP}}[\mu\nu ]\right)= \frac{1}{2}\sum_{\mu \in {\text{E}}\left(\mu \right)}|{{\text{c}}}_{\mu }-{{\text{c}}}_{\nu}|$$
  $$= \frac{1}{2}\left[ {\left| {4 - 1} \right|\left\{ {4\left( {\mathcalligra{r} + \mathcalligra{s} - 1} \right)} \right\} + \left| {3 - 1} \right|{\text{~}}\left\{ {4\left( {\mathcalligra{r}\mathcalligra{s} + \mathcalligra{r} + \mathcalligra{s} } \right)} \right\} + \left| {1 - 1} \right|\left\{ {4\left( {2\mathcalligra{r}\mathcalligra{s} - \mathcalligra{r} - \mathcalligra{s} } \right)} \right\}} \right]$$
  $$= 6\left(\mathcalligra{r}+\mathcalligra{s}-1\right)+4\left(\mathcalligra{r}\mathcalligra{s}+\mathcalligra{r}+{\mathcalligra{s}}\right)$$
  $${{\text{CIRR}}}_{{\text{t}}}\left({\text{InP}}[\mathcalligra{s}, \mathcalligra{r}]\right)=4\mathcalligra{r}, \mathcalligra{s}+10\mathcalligra{r}+10\mathcalligra{s}-6$$

## Numerical results and discussion

**Fig. 2:**
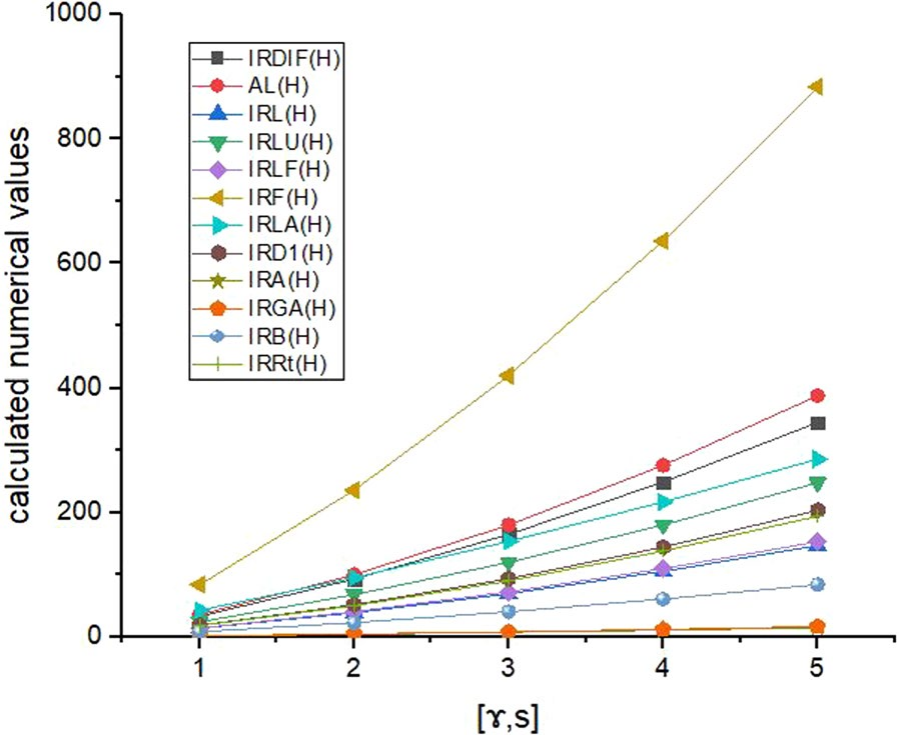
Graphical representation of Table 2

**Fig. 3:**
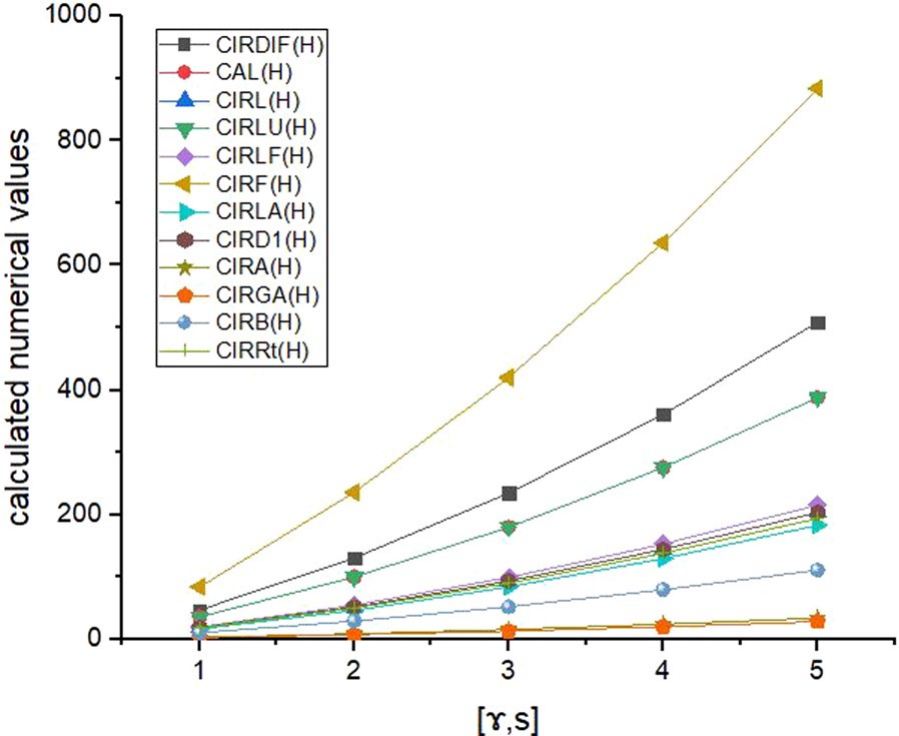
Graphical representation of Table 3

Here, we explored the indium phosphide $({\text{InP}})$, defined in Fig. 1, the moving parameters for this structure are $\mathcalligra{s}, \mathcalligra{r} \ge 1$. We have computed irregularity indices as well as reverse irregularity indices of indium phosphide ${\text{InP}}[\mathcalligra{s}, \mathcalligra{r}]$. The numerical values of the derived analytical expressions of degree-based irregularity indices and reverse irregularity indices are presented in Tables 2, 3, and the corresponding graph representations are shown in Figs. 2, 3 respectively. The comparison of these indices in Figs. 2, 3 depict that, IRF(H) and CIRF(H) have higher values as compared to other indices, it shows that these indices have high power of prediction of the physico-chemical properties of the molecular structure. Hence, the analytical expressions of these indices, in turn, referred to as tools for predicting several properties of molecular compounds in replacement of the laboratory experiments. In this regard, these indices are critical for capturing the molecular structure into a real number and predicting the important properties of chemical compounds. As a result, we believe that our these research results could be useful in predicting various properties of indium phosphide $({\text{InP}})$.

**Table 2:** Numerical table of irregularity indices associated with the structure of ${\text{InP}}[\mathcalligra{s}, \mathcalligra{r}]$ for different values of $\mathcalligra{s}, \mathcalligra{r}.$

$[\mathcalligra{s}, \mathcalligra{r}]$	$\left[\mathrm{1,1}\right]$	$\left[\mathrm{2,2}\right]$	$\left[\mathrm{3,3}\right]$	$\left[\mathrm{4,4}\right]$	$[\mathrm{5,5}]$
IRDIF (ℍ)	$33$	$93$	$165$	$249$	$345$
AL(ℍ)	$36$	$100$	$180$	$279$	$388$
IRL(ℍ)	$13.8624$	$38.8148$	$69.312$	$105.354$	$146.9408$
IRLU(ℍ)	$24$	$68$	$120$	$180$	$248$
IRLF(ℍ)	$14.4852$	$40.6272$	$72.426$	$109.8816$	$152.994$
IRF(ℍ)	$84$	$236$	$420$	$636$	$884$
IRLA(ℍ)	$42.6667$	$95.4667$	$153.6$	$217.0667$	$285.8667$
IRD1(ℍ)	$18.7284$	$51.7908$	$93.642$	$144.282$	$203.7108$
IRA(ℍ)	$1.5148$	$4.3728$	$7.574$	$11.1184$	$15.006$
IRGA(ℍ)	$1.5992$	$4.562$	$7.996$	$11.9012$	$16.2776$
IRB(ℍ)	$8.1172$	$22.9792$	$40.586$	$60.9376$	$84.034$
IRR_t_ (ℍ)	$18$	$50$	$90$	$138$	$194$

**Table 3:** Numerical table of reverse irregularity indices associated with the structure of ${\text{InP}}[\mathcalligra{s}, \mathcalligra{r}]$ for different values of $\mathcalligra{s}, \mathcalligra{r} .$

$[\partial ]$	$\left[\mathrm{1,1}\right]$	$\left[\mathrm{2,2}\right]$	$\left[\mathrm{3,3}\right]$	$\left[\mathrm{4,4}\right]$	$[\mathrm{5,5}]$
${\text{CIRDIF}}\left(\partial \right)$	$47$	$130.3333$	$235$	$361$	$508.3333$
CAL($\partial$)	$36$	$100$	$180$	$276$	$388$
CIRL($\partial$)	$18.7284$	$51.7908$	$93.642$	$144.282$	$203.7108$
CIRLU($\partial$)	$36$	$100$	$180$	$276$	$388$
CIRLF($\partial$)	$19.8564$	$54.9504$	$99.282$	$152.8512$	$215.658$
CIRF($\partial$)	$84$	$236$	$420$	$636$	$884$
CIRLA($\partial$)	$16.8$	$46.4$	$84$	$129.6$	$183.2$
CIRD1($\partial$)	$18.7284$	$51.7908$	$93.642$	$144.282$	$203.7108$
CIRA($\partial$)	$3.1432$	$8.7152$	$15.716$	$24.1456$	$34.004$
CIRGA($\partial$)	$2.618$	$7.2788$	$13.09$	$20.0516$	$28.1636$
CIRB($\partial$)	$10.4308$	$29.1488$	$52.154$	$79.4464$	$111.026$
${{\text{CIRR}}}_{{\text{t}}}\left(\partial \right)$	$18$	$50$	$90$	$138$	$194$

## Conclusion

Graph theory is a useful tool to model and characterize the molecular structures. In graph theory, the connections between atoms are represented as edges and the atoms themselves are represented as nodes. The distance between atoms, bond types, and 3D shapes of molecules can then be used to characterize the topology of the molecule. Furthermore, it can be used to calculate the topological descriptors of the molecule such as the degree-based and reverse degree-based irregularity toplogical indices. In this study, the 3D crystal structure of the InP is topologically modeled via partition of the edges, and derived closed form expressions for its irregularity indices. The numerical values of the derived analytical expressions of degree-based irregularity indices and reverse irregularity indices are then obtained and performed a comparatively analysis. The results show that, the topological indices IRF(H) and CIRF(H) have higher values as compared to other indices, it means that these indices have high power of prediction of the physico-chemical properties of indium phosphide $({\text{InP}})$. Hence, the derived analytical expressions of these indices, in turn, referred to as tools for predicting several properties of molecular compounds in replacing laborious laboratory experiments. In this regard, these indices are critical for capturing the molecular structure into a real number and predicting the important properties of chemical compounds. We believe that, these results will surely be helpful in investigating the QSPR/QSAR analysis as well as understanding the deep irregular behavior of the indium phosphide $({\text{InP}})$. In the near future, we aim to calculate the distance based and resistance distance based topological indices for certain 3D crystal structures.

## Data Availability

All data generated or analyzed during this study are included in this article.
